# HLTF promotes hepatocellular carcinoma progression by enhancing SRSF1 stability and activating ERK/MAPK pathway

**DOI:** 10.1038/s41389-023-00447-5

**Published:** 2023-01-20

**Authors:** Yanan Xu, Shanjia Ke, Shounan Lu, Chaoqun Wang, Zihao Li, Zhigang Feng, Hongjun Yu, Miaoyu Bai, Baolin Qian, Bing Yin, Xinglong Li, Yongliang Hua, Hongchi Jiang, Yong Ma

**Affiliations:** 1 Key Laboratory of Hepatosplenic Surgery, Ministry of Education, the First Affiliated Hospital of Harbin Medical University, Harbin, China; 2 Department of Minimal Invasive Hepatic Surgery, the First Affiliated Hospital of Harbin Medical University, Harbin, China; 3 The First Department of General Surgery, Affiliated Hospital of Inner Mongolia Minzu University, Tongliao, China; 4 Department of Pediatric Surgery, the First Affiliated Hospital of Harbin Medical University, Harbin, China

**Keywords:** Oncogenes, Liver cancer

## Abstract

Helicase-like transcription factor (HLTF) has been found to be involved in the progression of several tumors, but the role of HLTF in hepatocellular carcinoma (HCC) progression has not been studied. Here, our study explored the underlying mechanism of HLTF in HCC progression for the first time. Database analysis and clinical sample examination indicated that HLTF was upregulated in HCC tissues and was related to poor clinicopathological features in patients. Upregulation of HLTF accelerated the growth and metastasis of HCC cells both in vitro and in vivo. Bioinformatics analysis and subsequent experiments revealed that ERK/MAPK signaling pathway activation was vital to HLTF-mediated proliferation and metastasis in HCC cells. Moreover, HLTF was demonstrated to interact with SRSF1 and contribute to its protein stability to activate the ERK/MAPK signaling pathway and enhance HCC growth and metastasis. In addition, miR-511-5p was expressed at a low level in HCC tissues, was negatively correlated HLTF, and regulated HLTF expression. Our study shows that HLTF plays an oncogenic role in HCC progression and provides a novel biomarker and therapeutic target for the diagnosis and treatment of HCC.

## Introduction

According to global cancer statistics in 2020, there were approximately 906,000 new cases of liver cancer and 830,000 deaths; its prevalence rate ranks fifth among cancers [1]. Hepatocellular carcinoma (HCC) is the predominant pathological type of primary liver cancer, accounting for approximately 75–85% of cases [2]. Although surgery is the most effective treatment for HCC at present, HCC treatment still has many challenges because of its hidden onset, delayed diagnosis, strong invasiveness and high recurrence rate [3]. As a multikinase inhibitor, sorafenib is the first targeted drug approved for the treatment of advanced HCC patients, making molecular targeted therapy promising [4]. Therefore, we need to explore the molecular mechanism of HCC to search for new targets for the treatment of HCC.

Helicase-like transcription factor (HLTF), belonging to the SWI/SNF family, is involved in tumor progression in two ways, either epigenetic silencing by DNA methylation or overexpression [5]. Moinova et al. first observed *HLTF* promoter methylation in colon cancer [6]. Hibi et al. confirmed that the loss of HLTF gene expression was accompanied by *HLTF* promoter methylation in primary colon cancer, and inactivation could occur at the early stage in the tumorigenic pathway. Treatment with DNA methylation inhibitors can restore the activity of the HLTF gene and slow the rate of tumor progression [7]. *HLTF* promoter methylation is found more frequently in patients with family histories of gastric cancer in primary gastric cancer; 70–90% of early-stage cases in which the patient had a family history exhibited aberrant methylation of *HLTF. HLTF* methylation may play an important role in the early stage of gastric cancer in patients with family histories, and could be a susceptibility marker of gastric cancer risk in individuals with a family history [8]. HLTF has been identified as a tumor suppressive biomarker that is methylated in non-small cell lung cancer and hypermethylation of HTLF is associated with poor survival [9]. Nevertheless, in the experimental model of estrogen-induced renal carcinogenesis, HLTF expression was detected at the early stage of tumor progression, indicating that the activation of the HLTF gene is related to the initial steps of carcinogenesis [10]. HLTF contributes to radiation resistance by enhancing the DNA damage repair capacity in cervical cancer, while miR-145 overexpression can enhance the radiosensitivity of cervical cancer cells in vivo and in vitro. MiR-145 targets HLTF mRNA, and its expression level is negatively correlated with that of HLTF in radiation resistant cervical cancer tissues [11, 12]. Capouillez et al. revealed that HLTF was upregulated in hypopharyngeal squamous cell carcinoma, and a high level of HLTF was associated with a poor prognosis in patients. HLTF could be used as an independent prognostic marker of tumor recurrence [13]. HLTF has a low methylation rate in HCC [14], and we found in a public database that HLTF is abnormally upregulated in HCC and is related to a poor prognosis in HCC patients. However, the role of HLTF in HCC progression and its underlying mechanism are unknown.

In this study, we performed bioinformatics analysis and molecular biological experiments to clarify the mechanism by which HLTF potentiates the growth and metastasis of HCC. HLTF was generally upregulated in HCC tissues, and the upregulation of HLTF was related to a poor prognosis in HCC patients. Upregulation of HLTF promoted the growth and metastasis of HCC cells by interacting with and stabilizing SRSF1 and activating the ERK/MAPK signaling pathway. HLTF was negatively regulated by miR-511-5p. HLTF could become a new target for HCC diagnosis, treatment and prognosis evaluation.

## Results

### HLTF is upregulated in HCC tissues and associated with worse prognosis

**Fig. 1:**
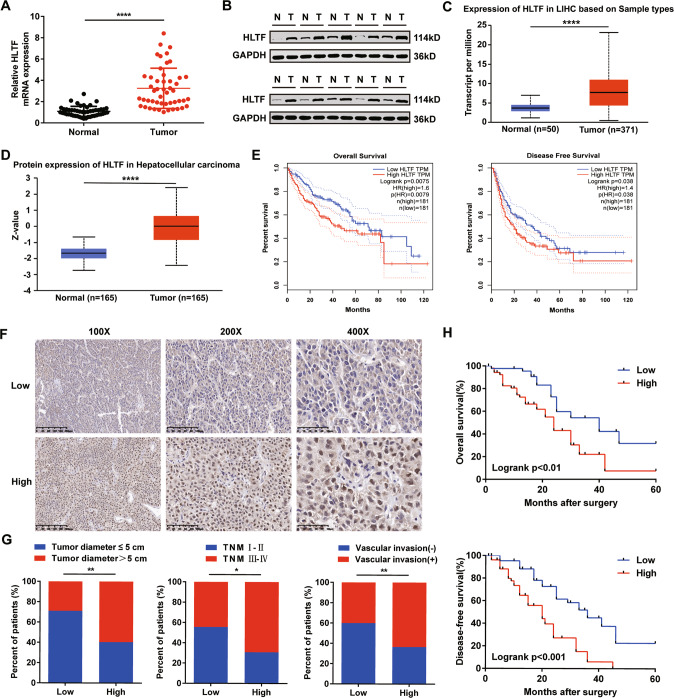
HLTF is upregulated in HCC and predicted poor prognosis. The expression levels of the HLTF mRNA (**A**) and protein (**B**) in HCC and adjacent tissues. The expression levels of HLTF mRNA (**C**) and protein (**D**) in the UALCAN database website. **E** Kaplan–Meier plot of overall survival and disease free survival of patients with HCC with high or low expression of HLTF in the TCGA-LIHC dataset from the GEPIA database website. **F** Representative images of HLTF IHC staining in HCC tissues. **G** Upregulation of HLTF was associated with poor clinicopathological feature, including tumor size, TNM stage, and venous invasion. **H** Kaplan–Meier curves of patients segregated by low or high expression of HLTF. *P < 0.05, **P < 0.01, ***P < 0.001, ****P < 0.0001. N normal; T tumor.

To explore HLTF expression in HCC tissues, we measured HLTF expression in HCC tissues and matched normal tissues by qRT‒PCR and western blotting. The results indicated that HLTF expression was upregulated in HCC tumor tissues (Fig. 1A, B). These assay results correspond with those in the UALCAN (http://ualcan.path.uab.EdU/) database website [15] (Fig. 1C, D). Additionally, analysis of the GEPIA database website (http://gepia.cancer-pku.cn/) [16] showed that high expression of HLTF in the TCGA-LIHC dataset was correlated with worse prognosis (Fig. 1E). Next, we performed immunohistochemical staining to detect HLTF expression and analyzed the correlations between HLTF expression and HCC clinicopathological characteristics in 97 patients (Fig. 1F). We found that high HLTF expression in HCC was positively related to tumor diameter, TNM stage and vascular invasion (Fig. 1G and Supplementary Table 4). We also found that patients with high HLTF expression had worse OS and DFS than those with low HLTF expression (Fig. 1H). Based on these results, HLTF is mostly increased in HCC, and high HLTF expression is closely correlated with a worse prognosis.

### HLTF enhances the proliferation of HCC cells

Using data in the TCGA-LIHC database, we analyzed HLTF expression levels via GSEA and found that high HLTF expression was related to the proliferation [17] and metastasis [18] of liver cancer (Supplementary Fig. 1A). Additionally, we assessed HLTF mRNA and protein expression in normal liver cells and several HCC cell lines, and we found that expression was higher in HCC cell lines than in normal liver cells (Supplementary Fig. 1B). Then, we evaluated the function of HLTF in HCC by transfecting the lentiviral vector expressing HLTF-specific shRNA into Huh7 and HepG2 cells to silence HLTF and transfecting the lentiviral vector encoding HLTF into HCCLM3 and Hep3B-cell lines to upregulate HLTF (Supplementary Fig. 1C, D).

**Fig. 2:**
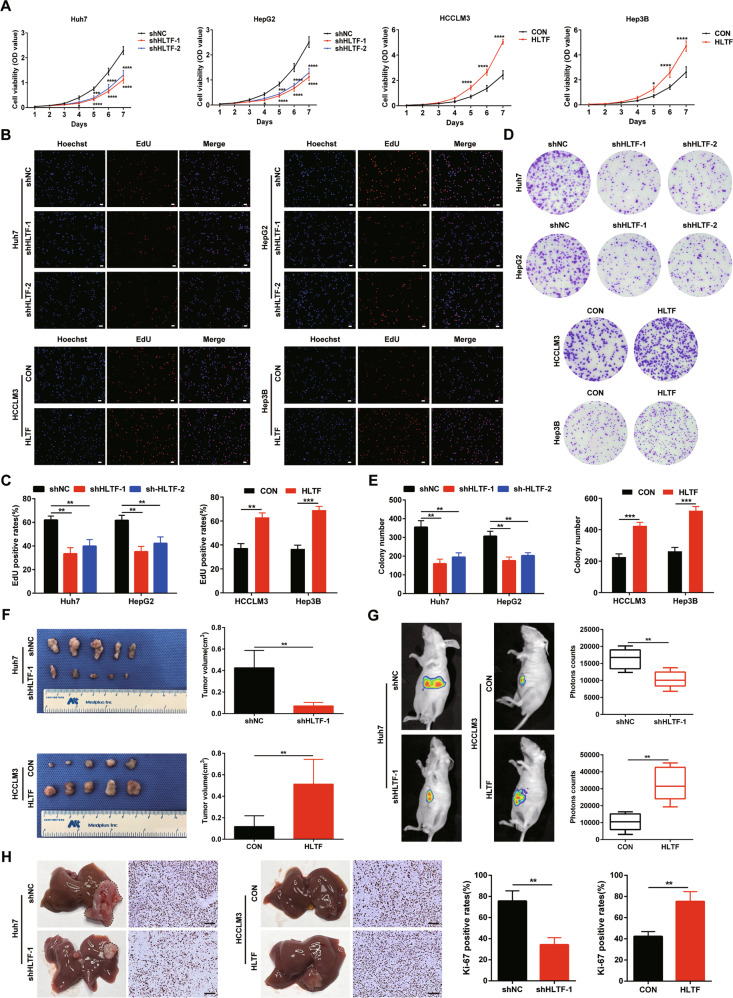
HLTF promotes the proliferation of HCC cells. **A** CCK-8 assay was used to evaluate the proliferation of indicated HCC cell lines. **B**, **C** Representative images and statistical analysis of the EdU assay of HCC cells. Scale bars: 200 μm. **D**, **E** Representative images and statistical analysis of the colony formation assay of indicated HCC cells. **F** Subcutaneous xenografts derived from indicated HCC cell lines and tumor volume analysis of subcutaneous xenografts (*n* = 5/group). **G** Representative images and statistical analysis of liver samples from liver orthotopic models (*n* = 5/group). **H** Representative images and statistical analysis of IHC staining for Ki-67 in liver orthotopic models. Scale bars: 200 μm. Experiments were done three times. Data are presented as means ± SD. *P < 0.05, **P < 0.01, ***P < 0.001, ****P < 0.0001.

We conducted a CCK-8 assay to evaluate the effect of HLTF on the viability and proliferation of HCC cells. Silencing HLTF dramatically decreased the viability of HCC cells and reduced their proliferation ability, whereas HLTF overexpression significantly increased the viability and enhanced the proliferation of HCC cells (Fig. 2A). The EdU results showed that there were fewer EdU-positive cells in the shHLTF groups than in the control group, but there were more EdU-positive cells in the HLTF overexpression group (Fig. 2B, C). Colony formation assays also suggested that HLTF enhances the proliferation of HCC cells (Fig. 2D, E). Similarly, the tumor volume was markedly reduced in the Huh7-shHLTF-1 group compared with the corresponding control group; accordingly, the tumor volume of the HCCLM3-HLTF group was markedly increased compared with that in the HCCLM3-CON group (Fig. 2F). Moreover, these results were also consistent with those we obtained from the orthotopic xenograft model (Fig. 2G). Furthermore, IHC staining revealed a reduction in Ki-67 staining in HLTF-silenced xenograft tumors and an increase in HLTF-overexpressing tumors compared with control tumors (Fig. 2H). Based on our findings, HLTF facilitates HCC proliferation in vitro and tumorigenesis in vivo.

### HLTF promotes the migration and invasion of HCC cells

**Fig. 3:**
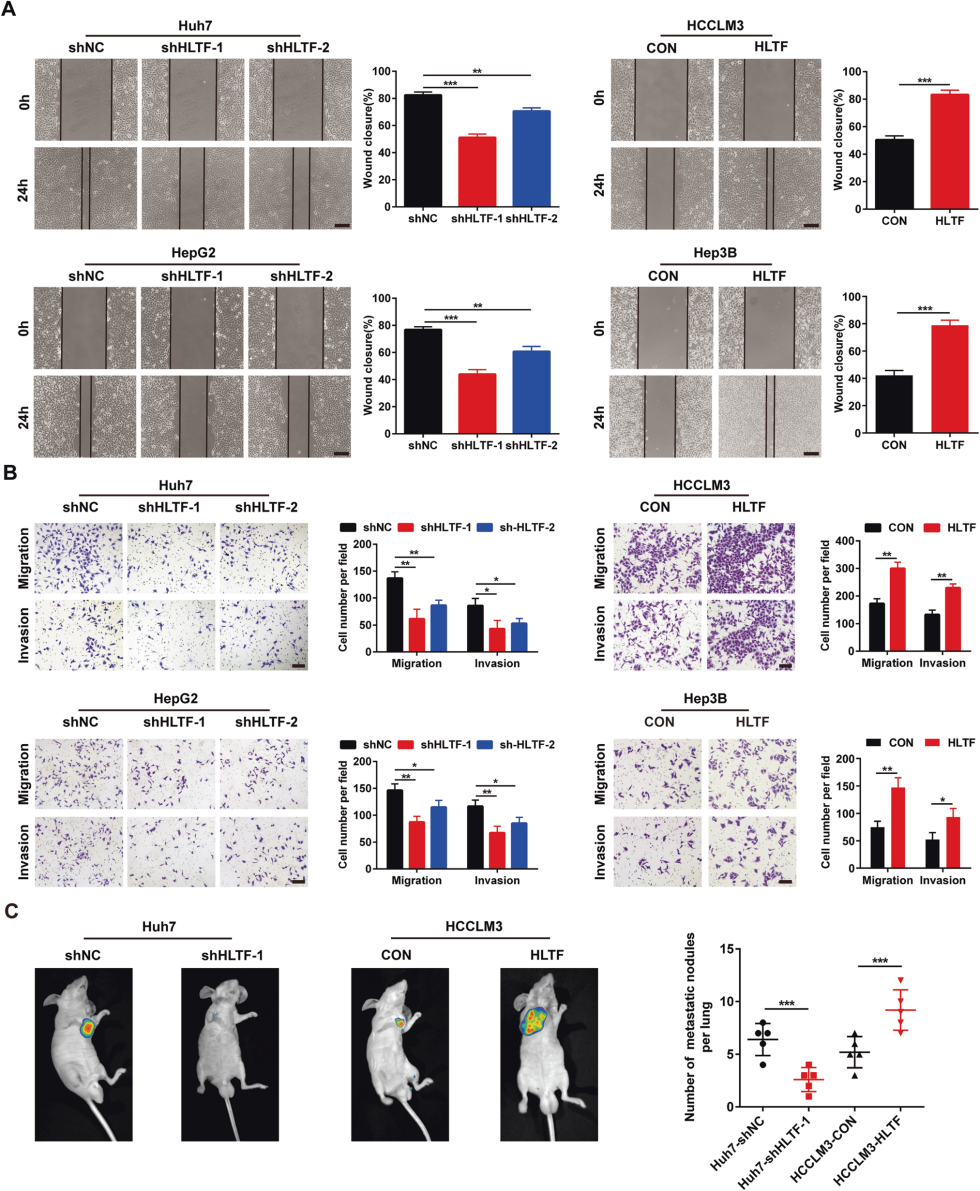
HLTF promotes the migration and invasion of HCC cells. **A** Representative images and statistical analysis of the wound-healing assay of HCC cells. Scale bars: 100 μm. **B** Representative images and corresponding statistical analysis of Transwell migration and invasion assays. Scale bars: 100 μm. **C** Representative bioluminescence images of lung metastasis model and statistical analysis of the number of metastatic nodules (*n* = 5/group). Experiments were done three times. Data are presented as means ± SD. *P < 0.05, **P < 0.01, ***P < 0.001.

In addition to its effect on proliferation, HLTF also regulates the metastasis of HCC (Supplementary Fig. 1A). Wound-healing assays indicated that the cell migratory ability was repressed when HLTF was downregulated, but this ability was enhanced when HLTF was upregulated (Fig. 3A). Transwell assays with chambers uncoated and coated with Matrigel showed that the migratory and invasive behaviors of HCC cells were obviously weakened after HLTF silencing, while HLTF overexpression enhanced both behaviors (Fig. 3B). We injected stably transfected HCC cells into the tail vein of mice to observe the effect of HLTF on metastasis. We found that the number and size of lung metastatic nodules were reduced in the HLTF-silenced group in comparison with the control group, while these were increased in the HLTF-overexpressing group (Fig. 3C). In summary, HLTF promotes HCC cell migration and invasion in vitro and metastasis in vivo.

### ERK/MAPK pathway is essential for HLTF-induced proliferation and metastasis in HCC

**Fig. 4:**
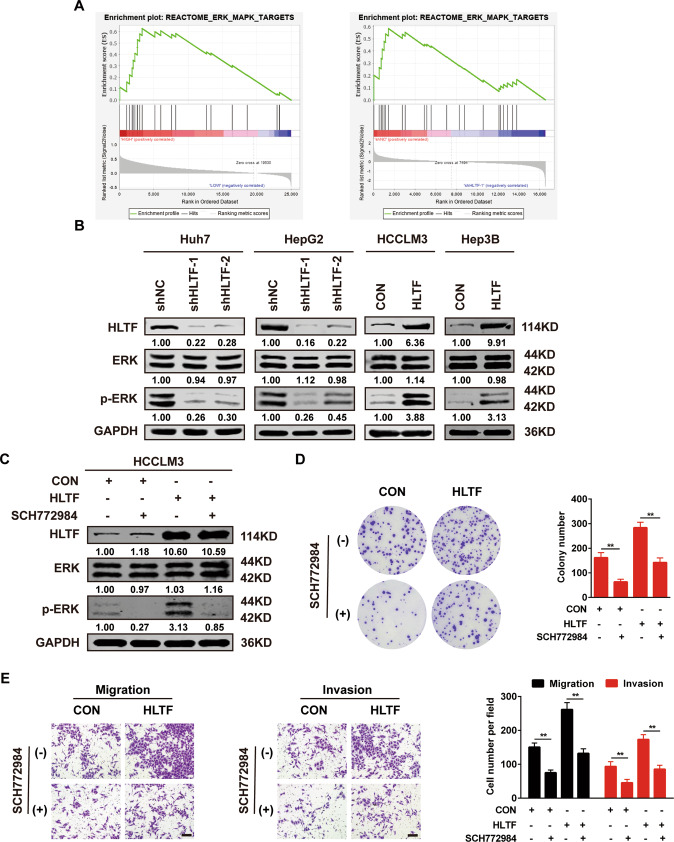
ERK/MAPK pathway is involved in HLTF-induced proliferation and invasion in HCC. **A** The result of GSEA showed that ERK/MAPK targets (REACTOME) signature was significantly enriched with HLTF high expression. **B** The protein expression of ERK and p-ERK were detected after HLTF silencing or overexpression. **C** The expression levels of HLTF, ERK and p-ERK in HCCLM3 cell line were detected by western blotting after SCH772984 treatment (10 μM). **D** Representative images and statistical analysis of colony formation assay after SCH772984 treatment (10 μM). **E** Representative images and statistical analysis of Transwell assays after SCH772984 treatment (10 μM). Scale bars: 100 μm. Experiments were done three times. Data are presented as means ± SD. **P < 0.01.

To clarify the molecular mechanism by which HLTF regulates the proliferation and metastasis of HCC, we performed bioinformatics analysis and found that the ERK/MAPK pathway was significantly enriched when HLTF was upregulated (Fig. 4A). Subsequently, this result was confirmed by western blotting in HLTF-knockdown and HLTF-overexpressing cell lines (Fig. 4B). Therefore, we conducted experiments to investigate whether HLTF regulates HCC cell proliferation and metastasis via the ERK/MAPK signaling pathway. SCH772984, a specific small molecule inhibitor of ERK1/2 [19, 20], reversed ERK/MAPK pathway activation in HCCLM3-HLTF cells (Fig. 4C). The colony formation assays showed that the number of clones formed was dramatically decreased when HCCLM3-HLTF cells were treated with SCH772984 (Fig. 4D). Additionally, treatment with SCH772984 prevented HCCLM3-HLTF cells from migrating and invading (Fig. 4E). These results show that the ERK/MAPK pathway is of vital importance in HLTF-induced HCC development.

### HLTF interacts with SRSF1 and promotes its protein stability to regulate the ERK/MAPK signaling pathway

**Fig. 5:**
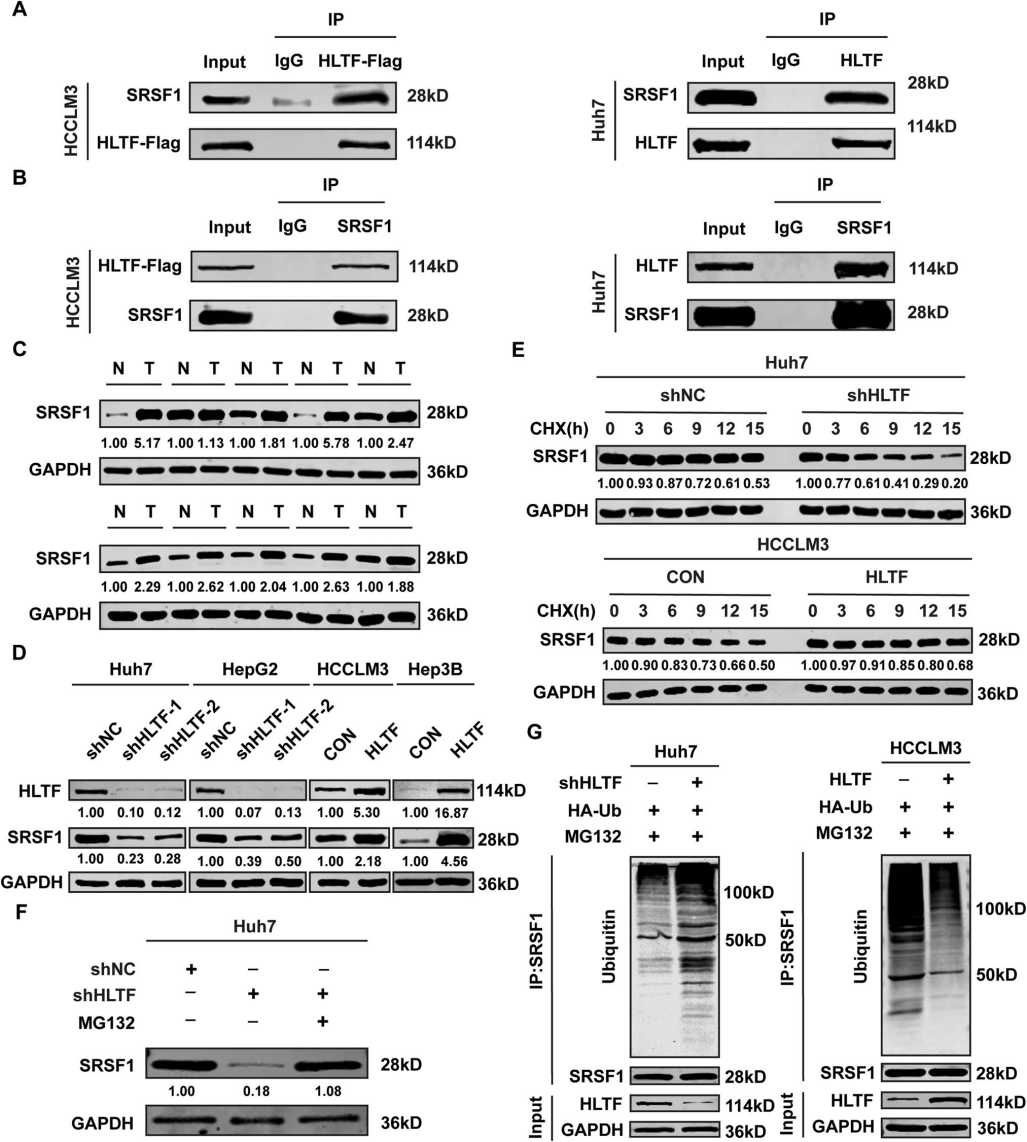
HLTF interacts with SRSF1 and promotes its stability. **A** Coimmunoprecipitation indicated that HLTF could interact with SRSF1 in HCCLM3 and Huh7 cells. **B** Coimmunoprecipitation indicated that SRSF1 could interact with HLTF in HCCLM3 and Huh7 cells. **C** The expression level of SRSF1 was determined by western blotting in HCC tissues and matched normal tissues. **D** Western blot analysis for SRSF1 in HLTF silencing or overexpressing HCC cells. **E** SRSF1 protein levels in Huh7 cells by downregulating HLTF or in HCCLM3 cells by upregulating HLTF at the indicated times after CHX (50 μg/ml) addition. **F** The protein levels of SRSF1 in HLTF silencing cells treated with MG132 (20 μM). **G** The ubiquitination of SRSF1 in HLTF silencing cells and the transfected cells were treated with MG132 (20 μM for 24 h) prior to harvest; the ubiquitination of SRSF1 by HLTF overexpression in HCCLM3 cells. Experiments were done three times. N normal; T tumor.

To explore the underlying mechanism of the HLTF-affected ERK/MAPK signaling pathway, we analyzed the protein interaction profile of HLTF in HCCLM3-HLTF cells by immunoprecipitation/mass spectrometry and obtained some candidate proteins (Supplementary Table 5). We identified a coprecipitated protein, SRSF1, by coimmunoprecipitation (Fig. 5A, B). Then, we measured the expression of SRSF1 in HCC tissues and matched normal tissues and found that the expression of SRSF1 was much higher in HCC tissues than in normal tissues (Fig. 5C). Western blotting was performed to test the protein levels of HLTF and SRSF1 in HCC cells. The results showed that the variation trend of SRSF1 was consistent with that of HLTF, decreasing when HLTF was downregulated, and vice versa (Fig. 5D). However, the RNA-seq results showed no obvious difference in SRSF1 mRNA levels between Huh7-shHLTF-1 cells and Huh7-shNC cells (data not shown). According to the results above, HLTF upregulates SRSF1 at the protein level instead of at the mRNA level. Hence, we considered that HLTF may mediate SRSF1 expression via posttranslational regulation. To investigate whether HLTF could enhance the protein stability of SRSF1, we treated cells with cycloheximide (CHX, an inhibitor of protein synthesis) and assessed the effect of HLTF on the SRSF1 degradation rate by western blotting at various time points. When HLTF expression was silenced in Huh7 cells, SRSF1 protein stability was significantly decreased, whereas HLTF overexpression dramatically slowed the rate of SRSF1 protein degradation in HCCLM3 cells (Fig. 5E). Subsequently, we investigated the pathways by which HLTF mediates the degradation of SRSF1 protein. We found that MG132 (an inhibitor of the ubiquitin–proteasome pathway) protected the SRSF1 protein from degradation in HLTF knockdown cells (Fig. 5F). Furthermore, HLTF knockdown significantly enhanced the ubiquitination of SRSF1 in Huh7 cells in the presence of MG132; however, the ubiquitination of SRSF1 was significantly suppressed by HLTF overexpression in HCCLM3 cells (Fig. 5G). These results indicate that HLTF enhances SRSF1 protein stability by protecting SRSF1 from ubiquitination and subsequent proteasomal degradation.

Next, we silenced SRSF1 in the HCCLM3 cells and determined the transfection efficiency (Supplementary Fig. 2A). Subsequently, western blotting analysis showed that SRSF1-silencing interfered with the activation of the ERK/MAPK signaling pathway caused by HLTF overexpression (Supplementary Fig. 2B). In addition, silencing SRSF1 reversed the promotional effect on proliferation, migration and invasion mediated by HLTF overexpression in HCC cells (Supplementary Fig. 2C, D). In vivo, inhibiting SRSF1 decreased the volume of subcutaneous tumors (Supplementary Fig. 2E). Therefore, we propose that HLTF interacts with SRSF1 to regulate the ERK/MAPK signaling pathway and facilitate HCC progression.

### MiR-511-5p targets HLTF and inhibits HLTF-mediated proliferation and metastasis in HCC

An increasing number of studies have reported that miRNAs participate in various biological processes, including tumor progression, by regulating gene expression. Therefore, to investigate the causes of abnormal upregulation of HLTF in HCC, we accessed online public databases to identify miRNAs that could be upstream regulators of HLTF and screened six candidate miRNAs (Supplementary Fig. 3A). Only miRNA-511-5p was significantly downregulated and negatively correlated with HLTF in the TCGA-LIHC dataset (Supplementary Fig. 3B, C). We found the same results in our clinical tissues (Supplementary Fig. 3D, E). Similarly, miR-511-5p expression in the WRL68 cell line was higher than that in HCC cell lines (Supplementary Fig. 3F).

**Fig. 6:**
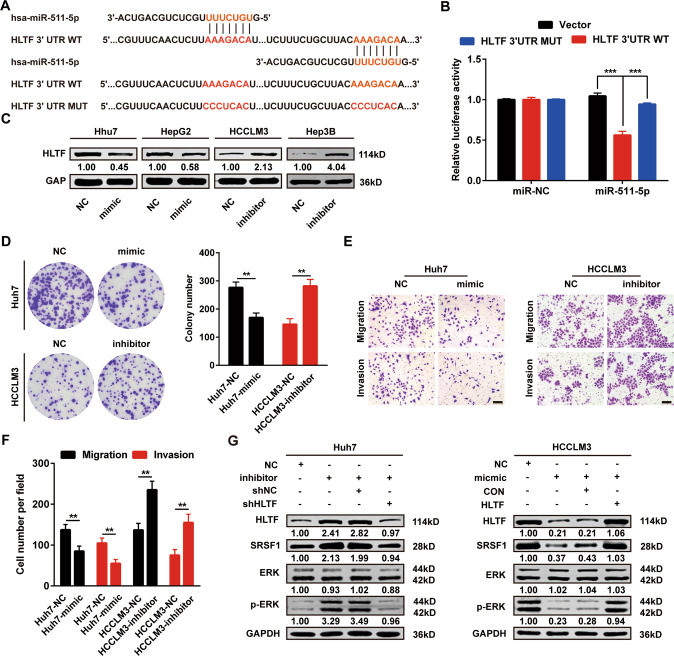
MiR-511-5p targets HLTF and inhibits HLTF-mediated proliferation and metastasis in HCC. **A** Binding sites between miR-511-5p and wild-type or mutant 3ʹ-UTRs sequences of HLTF mRNA. **B** Result of the luciferase reporter assay. Luciferase activity of 293T cell transfected with wild-type 3′-UTR was significantly suppressed by miR-511-5p overexpression. **C** The protein level of HLTF was decreased or promoted under the condition of miR-511-5p overexpression or silencing. **D** Representative images of colony formation assay and corresponding statistical analysis in indicated groups. **E**, **F** Representative images of Transwell migration and invasion assays and corresponding statistical analysis in indicated groups. **G** The expression of SRSF1 and the ERK/MAPK pathway was analyzed by western blot after transfecting with miR-511-5p inhibitor NC or miR-511-5p inhibitor in Huh7 cell, or miR-511-5p mimic NC or miR-511-5p mimic in HCCLM3 cell. Experiments were done three times. Data are presented as means ± SD. **P < 0.01, ***P < 0.001.

We then designed wild-type and mutant 3ʹ-UTRs of HLTF mRNA for the luciferase reporter assay (Fig. 6A), and the results showed that luciferase activity was remarkably suppressed by miR-511-5p-overexpression in the wild-type 3′-UTR group, but was not significantly altered in the mutant 3′-UTR group (Fig. 6B), suggesting that HLTF is a direct target of miR-511-5p. Next, in HCC cells, we upregulated or downregulated miR-511-5p with mimics or inhibitors, respectively, and showed by western blotting that HLTF expression was inversely correlated with miR-511-5p expression (Fig. 6C). Colony formation and Transwell assays suggested that miR-511-5p and HLTF had opposite effects on the proliferation and metastasis of HCC cells (Fig. 6D–F). Moreover, we performed rescue experiments to confirm that miR-511-5p participates in the HCC-promoting function of HLTF. These results revealed that knockdown of HLTF by the miR-511-5p inhibitors reversed the activation of the downstream molecule SRSF1 and the ERK/MAPK signaling pathway, and conversely, overexpression of HLTF by miR-511-5p mimics restored the inhibitory state of the downstream molecule SRSF1 and the ERK/MAPK signaling pathway (Fig. 6G). These results indicate that miR-511-5p negatively regulates HLTF, which in turn prevents HLTF-mediated proliferation and metastasis in HCC.

## Discussion

In recent years, driven by integrated multiomics analysis, a molecular classification system based on gene signature, metabolism, immunity, and chromosome spectrum has emerged for HCC, which provides a good basis for subtype-specific targeted HCC therapies and encourages us to more actively explore the molecular mechanisms of the occurrence and progression of HCC [21]. This study is the first to explore the role of HLTF in HCC.

In our study, by searching a public database website, we found that HLTF was abnormally upregulated in HCC and that the upregulation of HLTF was related to a poor prognosis. Subsequently, we used clinical specimens for further verification. The results revealed that compared with that in normal liver tissues, HLTF expression was significantly increased in tumor tissues, and HLTF expression was significantly correlated with clinicopathological characteristics and patient outcome. In vivo and in vitro experiments also confirmed that HLTF can promote the growth and metastasis of HCC cells. Therefore, HLTF is crucial to the occurrence and development of HCC.

Subsequently, we performed bioinformatics analysis and found that the ERK/MAPK pathway could be the key pathway through which HLTF promotes the progression of HCC. Further experiments confirmed that HLTF affected the levels of p-ERK in HCC cells; after transfected HLTF cells were treated with SCH772984, ERK/MAPK pathway activation and the tumor-promoting effects mediated by HLTF were suppressed. Thus, ERK/MAPK pathway activation is very important for HLTF-mediated induction of HCC cell proliferation and metastasis.

HLTF was found to promote HCC progression by activating the ERK/MAPK signaling pathway in our study; however, the specific activation mode requires further research. Mass spectrometry analysis and coimmunoprecipitation assays identified and verified the interaction between HLTF and SRSF1 in HCC cells. SRSF1 is a representative member of the SR protein family, a family of RNA binding proteins, and is the first member of this family to be identified as a proto-oncogene [22, 23]. It has been reported that SRSF1 is highly expressed in multiple tumor tissues [22, 24–26], and similarly, SRSF1 is upregulated in HCC tumor tissues. It has been confirmed that SRSF1 may promote the activation of the ERK/MAPK signaling pathway by increasing the levels of B-RAF mRNA and protein [27]. A recent study also showed that SRSF1 can regulate the splicing of SRA1 in HCC, thereby promoting the transcription of *CD44* and activating the ERK and AKT signaling pathways, and affecting the metastasis of HCC [28]. We further demonstrated that HLTF upregulates SRSF1 at the protein level rather than the mRNA level. The stability of SRSF1 protein was significantly reduced in HLTF knockdown HCC cells, but the ubiquitination of SRSF1 was enhanced in the presence of MG132, while the result was the opposite in HLTF-overexpressing cells. HLTF has been reported to act as an E3 ubiquitin ligase to promote the polyubiquitination of PCNA by forming a thioester-linked Ub chain, thus participating in postreplication DNA repair [29, 30]. Intriguingly, our results suggested that HLTF regulates SRSF1 protein stability in a manner independent of its E3 ligase function; specially, it protects SRSF1 from polyubiquitination and subsequent proteasome degradation.

*HLTF* promoter methylation often occurs in colorectal cancer and gastric cancer, but the incidence of *HLTF* promoter methylation in HCC is very low [7, 14]. Therefore, when exploring the upstream regulatory mechanism of abnormal expression of *HLTF* in HCC, we focused on miRNAs. Numerous studies have also confirmed that miRNAs play a key role in the activation of oncogenes and the inactivation of tumor suppressors, which is closely associated with tumor occurrence and development [31–34]. In our study, we found by a luciferase reporter assay that miR-511-5p, as the upstream regulator of HLTF, directly targets HLTF. It has been reported that miR-511-5p expression is markedly reduced in gastric and colorectal cancer and functions as a tumor suppressor [35, 36]. We also found that miR-511-5p was markedly downregulated in HCC, had a negative correlation with HLTF and interfered with the growth and metastasis of HCC mediated by HLTF.

In summary, we confirmed that HLTF is involved in the progression of HCC and enhances its growth and metastasis. HLTF plays a tumor-promoting role by activating the ERK/MAPK signaling pathway by increasing SRSF1 protein stability. In addition, miR-511-5p targets HLTF and negatively regulates HLTF-mediated proliferation and metastasis in HCC. These results provide new insight into HCC patient diagnosis, treatment and prognosis evaluation.

## Materials and methods

### Cell transfection

Lentiviral vectors for *HLTF* gene knockdown (Lv-shHLTF) and overexpression (Lv-HLTF) and empty vectors were manufactured by and obtained from HanBio (Shanghai, China). Oligonucleotides for inhibitors, mimics and negative controls were obtained from RiboBio Corporation (Guangzhou, China); h-SRSF1 single gene siRNA and negative control siRNA (si-NC) were also obtained from RiboBio Corporation (Guangzhou, China). All cell transfections were performed in accordance with the manufacturer’s guidelines. Information on specific sequences is provided in Supplementary Table 1.

### Western blotting

Tissues or cells were lysed with RIPA buffer supplemented with protease and phosphatase inhibitors, and proteins were harvested. Then, the proteins were separated on PAGE gels and electrotransferred onto NC membranes (BioTrace, New Zealand, USA). The NC membranes were blocked in 5% bovine serum albumin and sequentially incubated with primary antibodies and secondary antibodies (LI-COR Biosciences, Nebraska, USA). Protein expression was visualized by the Odyssey CLx Imaging System (LI-COR Biosciences). Information on the primary antibodies is listed in Supplementary Table 2.

### Real-time quantitative PCR (qRT‒PCR)

Total RNA was extracted using an RNA Miniprep Kit (Axygen Scientific, Inc., USA) according to the manufacturer’s guidelines and was reverse-transcribed into cDNA using a ReverTra Ace qPCR RT Kit (TOYOBO, Japan) or Bulge-Loop miRNA qRT‒PCR Starter Kit (RiboBio Corporation). Real-time PCR was conducted using FastStart Universal SYBR Green Master Mix (Rox) (Roche) or a Bulge-Loop miRNA qRT‒PCR Starter Kit (RiboBio Corporation) on an ABIPRISM 7500HT instrument (Applied Biosystems, NY, USA). The expression levels of mRNAs and miRNAs were normalized to those of GAPDH and U6, respectively, and were calculated by the 2^−ΔΔCt^ method. The complete primer sequences are provided in Supplementary Table 3.

### RNA sequencing

RNA sequencing was completed by JLX MEDICAL SCIENCES (Shanghai, China). Briefly, Huh7 cells transfected with shHLTF-1 or shNC were used to extract total RNA for quality control. After the quality of the total RNA samples was tested, the library was constructed and sequenced using the DNB-SEQ platform. NCBI Sequence Read Archive sequencing data were uploaded under accession number PRJNA802308.

### Gene set enrichment analysis (GSEA)

Gene set enrichment analysis version 4.0.3 (Broad Institute, USA) [37] was performed to explore the potential biological function and signaling pathways involved in HCC through HLTF. The c2.all.v7.4.symbols.gmt dataset obtained from the Molecular Signatures Database was used as the reference dataset, and gene sets with *P* < 0.05 and FDR < 0.25 were considered statistically significant. Data from The Cancer Genome Atlas (TCGA), including 371 HCC tissues from RNA-seq data downloaded from the SangerBox platform (http://www.sangerbox.com/tool), and RNA-seq data from HCC cell lines (shNC vs. shHLTF-1) were analyzed by GSEA.

### Coimmunoprecipitation assay

The protein lysates were prepared and incubated with anti-FLAG (Cell Signaling Technology), anti-HLTF (Proteintech), anti-SRSF1 (Proteintech) and IgG antibodies (Cell Signaling Technology) at 4 °C overnight with gentle rotation. Then, Protein A/G Plus-Agarose (Santa Cruz, USA) was added to the protein-antibody complexes, and they were incubated with shaking for 3 h at 4 °C. Immunoprecipitates were collected by centrifugation, and then the complexes were washed three times. Then, proteins were eluted from Protein A/G Plus-Agarose, resuspended in 3×SDS buffer and boiled at 100 °C for 5 min before western blotting. Information on the primary antibodies is listed in Supplementary Table 2.

### Ubiquitination assay

Cells were transfected with lentiviruses containing control, HLTF, or HLTF-specific shRNAs, along with HA-tagged ubiquitin and treated with 20 μM MG132 for 24 h to block proteosomal degradation. The protein lysates were prepared and incubated with anti-SRSF1 (Proteintech). Then, Protein A/G Plus-Agarose (Santa Cruz, USA) was added to the protein-antibody complexes, and they were incubated with shaking for 3 h at 4 °C. Immunoprecipitates were collected by centrifugation, after which the complexes were washed three times. Then, proteins were eluted from Protein A/G Plus-Agarose, resuspended in buffer and boiled at 100 °C for 5 min before western blotting. Information on the primary antibodies is listed in Supplementary Table 2.

### Luciferase reporter assay

Cells were seeded in 24-well plates and cotransfected with the specific plasmids and pRL-TK Renilla. After culturing for 48 h, luciferase activity was detected by a dual-luciferase reporter assay kit (Promega, Madison, WI, USA). All operations were performed in accordance with the manufacturer’s guidelines.

### Statistical analysis

Statistical analyses were performed using GraphPad Prism 6.0 software (San Diego, USA). Data are presented as the mean values ± SDs. Statistics were determined between groups using Student’s t test or one-way ANOVA. Correlations were evaluated using the Spearman method. Kaplan–Meier statistics were used to evaluate overall survival (OS) and disease-free survival (DFS), and log-rank tests were used to determine significance. *P < 0.05, **P < 0.01, ***P < 0.001 and ****P < 0.0001 were considered statistically significant.

## Supplementary information

Supplementary file

## Data Availability

All data generated or analyzed during this study are available from the corresponding author on reasonable request.
