# Supplementary material for: HLTF promotes hepatocellular carcinoma progression by enhancing SRSF1 stability and activating ERK/MAPK pathway
Source: Oncogenesis. 2023 Jan 20;12(1):2. doi: 10.1038/s41389-023-00447-5 (PMC9859789; doi:10.1038/s41389-023-00447-5)
Supplement: Supplementary file 1 — Supplementary file [file 41389_2023_447_MOESM1_ESM.pdf]

Supplementary materials and methods

Cell lines

Huh7, HepG2, HCCLM3, and Hep3B cells were purchased from the Chinese Academy of Science (Shanghai, China). The normal liver cell line WRL68 was purchased from AcceGen (Fairfield, USA). Human HCC cell lines were cultured in Dulbecco's Modified Eagle Medium (Gibco, USA), and the normal liver cell line was cultured in RPMI 1640 (Gibco, USA). All culture medium was supplemented with 10% foetal bovine serum (FBS, Gibco, USA) and 1% penicillin–streptomycin (HyClone, USA). All cell lines were incubated at 37 °C in an incubator containing 5% CO2.

Human tissues

There were two separate cohorts consisting of 146 HCC patients in our study (cohort 1, n = 49; cohort 2, n = 97). HCC tissues and matched normal liver tissues were collected at the First Affiliated Hospital of Harbin Medical University from 2010 to 2018 from patients who underwent hepatectomy. Ethical approval was obtained from the Ethics Committee of the First Affiliated Hospital of Harbin Medical University, and informed consent was obtained from each patient.

Immunohistochemistry (IHC)

Tissue sections were deparaffinized, rehydrated, antigen repaired and then blocked in normal goat serum. After that, the sections were incubated with primary antibodies overnight at 4 °C. The next day, the sections were incubated with the secondary antibody (Vector Lab, Burlingame, CA) for one hour and then ABC reagent (Vector Lab) for half an hour at room temperature. Finally, the sections were stained with

diaminobenzidine (Vector Lab) and counterstained with hematoxylin (Sigma, Missouri, USA). Information on the primary antibodies is listed in Supplementary Table 2.

CCK-8 assay

Stably transfected cells were seeded in each well of a 96-well plate and incubated at 37 °C under 5% CO2 for attachment. Cell viability was determined by using a CCK-8 assay (Dojindo, Japan) at the indicated time points according to the manufacturer's instructions.

Colony formation assays

For colony formation assays, 500-800 cells were plated into each dish and cultured for 14 days. Then, the colonies were fixed with methanol and stained with 0.1% crystal violet to visualize the colonies.

EdU assay

An EdU assay kit (Beyotime, Shanghai, China) was used to analyze cell proliferation. All procedures were performed according to the manufacturer's instructions. Finally, the images were visualized under a fluorescence microscope.

Wound-healing assay

Stably transfected HCC cells were cultured overnight and allowed to grow to confluence, after which a straight wound was generated by gently scratching the cells with a sterile 200 μl pipette tip. The cells were washed three times with PBS and then cultured with medium containing 1% FBS. Wound closure was captured at 0h and 24h using a microscope.

Transwell assay

The migration and invasion capability *in vitro* was measured using 24-well plates not precoated or precoated with Matrigel (Corning, NJ, USA) on the membranes. In the upper chamber, we seeded 2-4 × 10^4 cells suspended in serum-free medium, and we filled the lower chamber with medium containing 20% FBS. After incubation at 37 °C for 24-48 hours, the cells were fixed with methanol for 15 minutes, stained with crystal violet for 20 minutes and counted under a microscope.

Animal studies

Male BALB/c nude mice, aged 4-6 weeks, were obtained from Charles River Lab Animal Center (Beijing, China) and raised in a specific pathogen-free facility. For the subcutaneous xenograft tumor model, 3×10^6 HCC cells suspended in 150 μl PBS were injected subcutaneously into the flanks of mice (n=5/group). After 4 weeks, the mice were killed, and the xenograft tumors were completely resected. The tumor volume was calculated by the following formula: $V=W^2\times L/2$. The excised subcutaneous xenograft tumor was cut into 1 mm^3 cubes and transplanted into the hepatic lobe of the nude mice to establish the orthotopic xenograft tumor model. The mice were sacrificed at the sixth week, and the tumors were collected.

The pulmonary metastasis nude mouse model was developed as follows: 3×10^6 cells dissolved in 150 μl PBS were injected into the tail vein of the mice (n=5/group). After six weeks, the nude mice were measured using the Berthold NIGHTOWL imaging system and sacrificed to collect their lungs. All experiments involving animals were conducted according to the standard protocols of the Institutional Animal Care and Use Committee of Harbin Medical University.

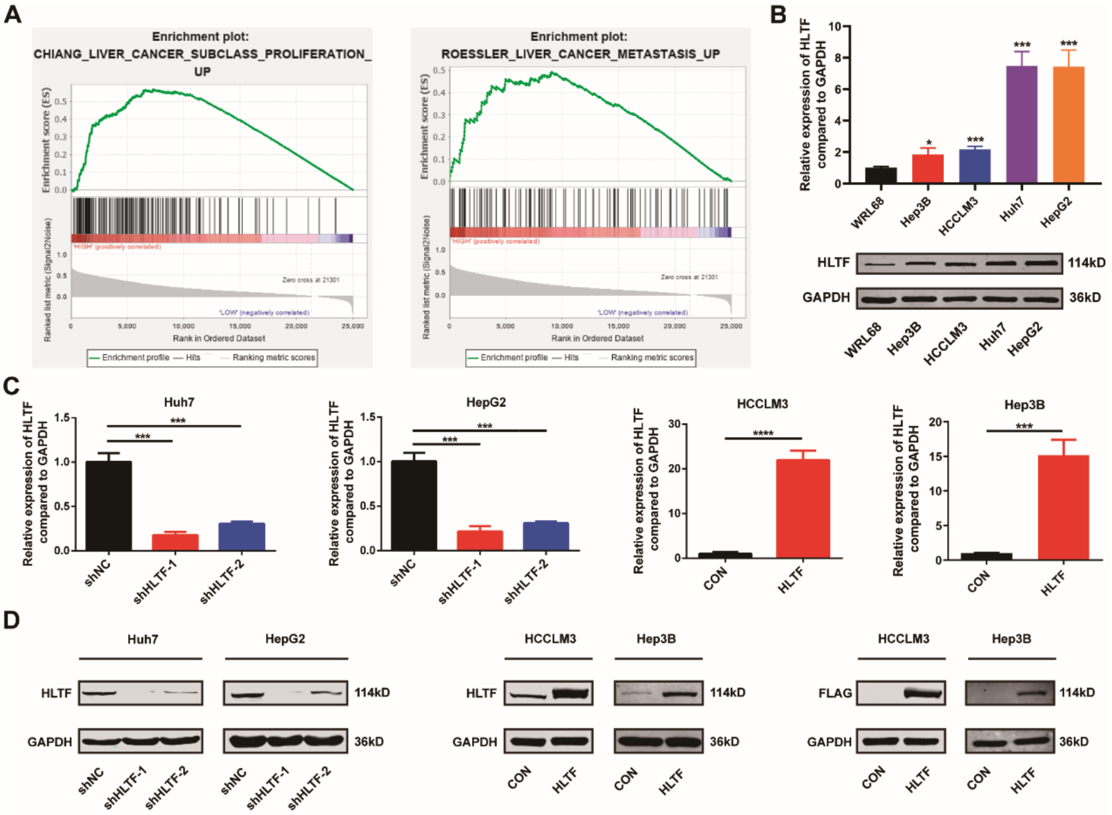

Supplementary Fig. 1A Gene set enrichment analysis showed that HLTF was associated with liver cancer proliferation and metastasis. **B** The expression levels of the HLTF mRNA and protein in normal liver cell line and HCC cell lines. **C** qRT–PCR analysis of the transfection efficiency of HLTF in HCC cell lines. **D** Western blot analysis of the transfection efficiency of HLTF in HCC cell lines. Data are presented as means ± SD. *P<0.05, **P<0.01, ***P<0.001, ****P<0.0001.

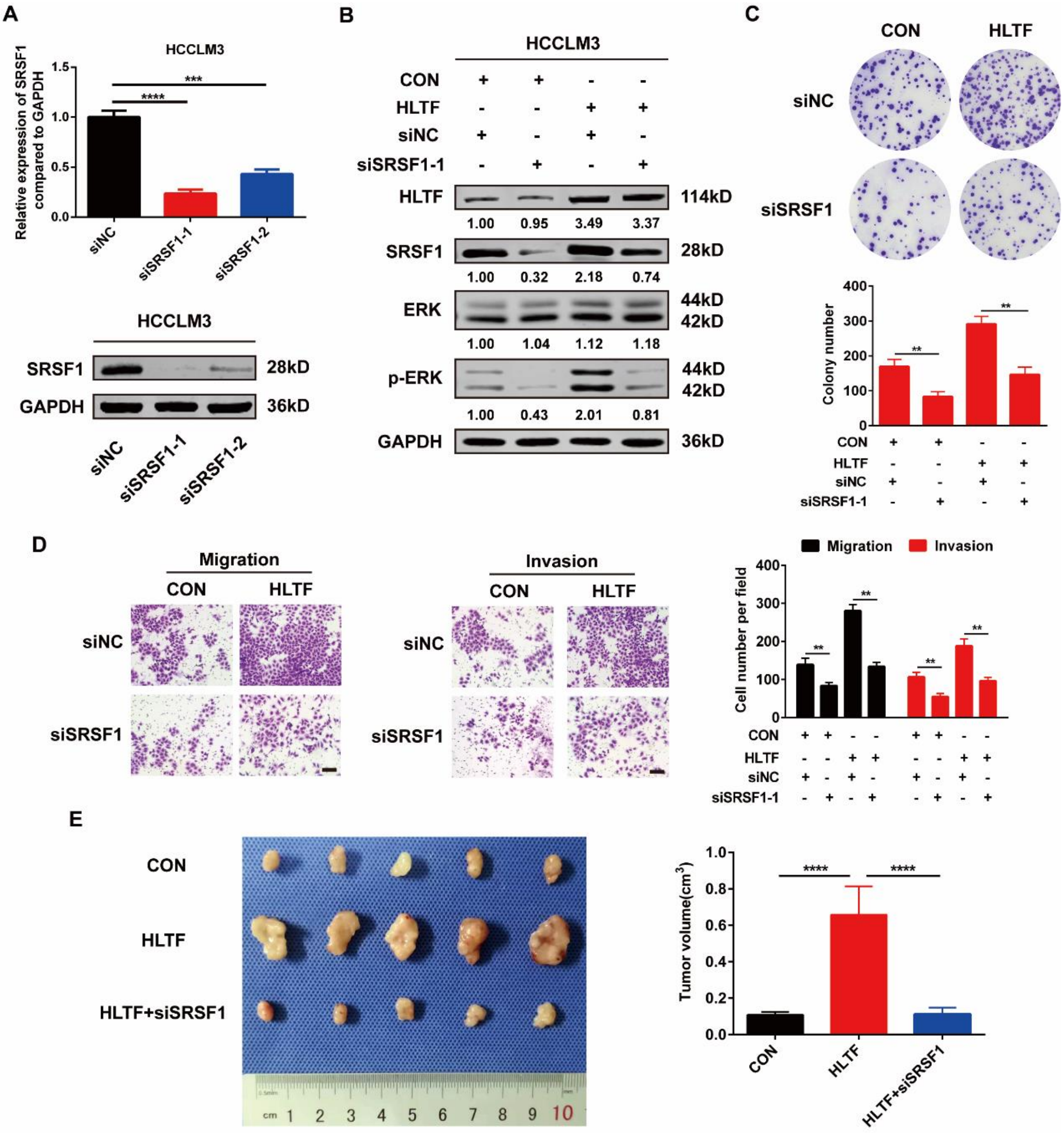

Supplementary Fig. 2A The expression levels of the SRSF1 mRNA and protein after si-SRSF1 transfection into HCCLM3 cell line. **B** Knockdown of SRSF1 interfered with the activation of the ERK/ MAPK signaling pathway caused by HLTF overexpression. **C** Representative images and statistical analysis of colony formation assay after si-SRSF1 transfection. **D** Representative images and statistical analysis of Transwell assays after si-SRSF1 transfection. Scale bars: 100 μm. **E** Subcutaneous xenografts images and tumor volume analysis of subcutaneous xenografts after si-SRSF1 transfection. (n=5/group). Experiments were done three times. Data are presented as

means ± SD. **$P<0.01$, ***$P<0.001$, ****$P<0.0001$.

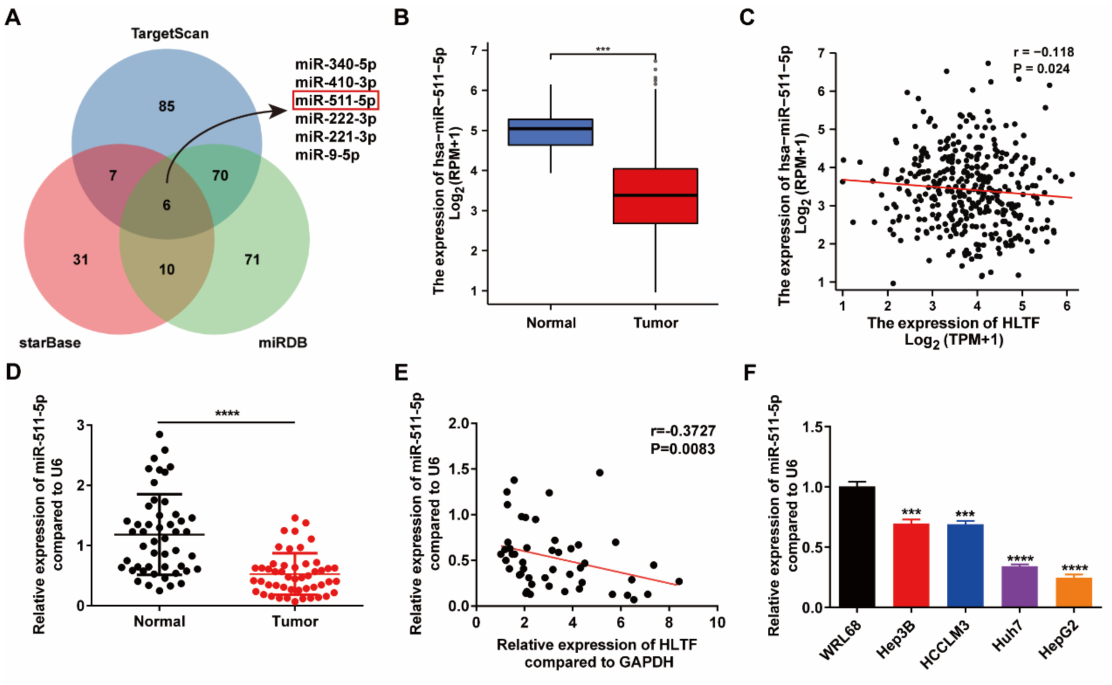

Supplementary Fig. 3A Six candidate miRNAs were screened from the Targetscan, Starbase and miRDB databases. **B** MiR-511-5p expression levels in tumor and normal samples from the TCGA-LIHC database. **C** Correlation analysis of the relative expression showed negative correlation between HLTF and miR-511-5p in the TCGA-LIHC database. **D** The expression levels of the miR-511-5p in tumor and normal tissues of 49 HCC patients. **E** Correlation analysis of the relative expression showed negative correlation between HLTF and miR-511-5p in 49 HCC patients. **F** Relative expression of miR-511-5p in the normal liver cell line WRL68 and HCC cell lines. Data are presented as means ± SD. ***P<0.001, ****P<0.0001.

Supplementary table 1 Target sequences information

	Target sequences (5'-3')
shHLTF-1	TAAGAAGGTAAGTATGGCAAC
shHLTF-2	TTTGTGATGATAACTTCTTGC
siSRSF1-1	CCAACAAGATAGAGTATAA
SiSRSF1-2	GGAAAGAAGATATGACCTA

Supplementary table 2 Antibody information

Antibody	Host	Product number	Company	Dilution
HLTF	Rabbit	ab183042	Abcam	1:1000(WB)
				1:100(IHC)
		14286-1-AP	Proteintech	4.0ug for IP and 1:1000 for WB
SRSF1	Rabbit	12929-2-AP	Proteintech	1:1000(WB)
				4.0ug for IP and 1:1000 for WB
ERK	Rabbit	#4695	Cell Signaling Technology	1:1000(WB)
p-ERK	Rabbit	#4370	Cell Signaling Technology	1:1000(WB)
FLAG	Rabbit	#14973	Cell Signaling Technology	1:1000(WB)
				1:50(IP)
Ubiquitin	Mouse	sc-8017	Santa Cruz Biotechnology	1:1000(WB)
Ki-67	Mouse	#9449	Cell Signaling Technology	1:500(IHC)
GAPDH	Mouse	60004	Proteintech	1:10000(WB)

Supplementary Table 3 Primer information

Gene Name	Sequence (5'-3')
HLTF	F: CTAAAGGCAGGGCGAAAG
	R: CGATCAGGACCATAATAAACAT
SRSF1	F: CCGCAGGGAACAACGATTG
	R: GCCGTATTTGTAGAACACGTCCT
GAPDH	F: CATGAGAAGTATGACAACAGCCT
	R: AGTCCTTCCACGATACCAAAGT
Bulge-Loop hsa-miR-511-5p Primer Set	RiboBio Corporation
Bulge-Loop U6 qPCR Primer Set	RiboBio Corporation

Supplementary Table 4 Relationship between HLTF expression and clinicopathologic features of HCC patients (n = 97)

Features	HLTF expression		P value
	Low (n=45)	High (n=52)	
Age, n (%)			0.995
≤60	25 (25.8%)	30 (30.9%)	
＞60	20 (20.6%)	22 (22.7%)	
Gender, n (%)			0.114
Male	26 (26.8%)	39 (40.2%)	
Female	19 (19.6%)	13 (13.4%)	
AFP (ug/L), n (%)			0.375
≤400	30 (30.9%)	29 (29.9%)	
＞400	15 (15.5%)	23 (23.7%)	
HBV infection, n (%)			0.395
Yes	29 (29.9%)	28 (28.9%)	
No	16 (16.5%)	24 (24.7%)	
Tumor diameter (cm), n (%)			**0.005**
≤5	32 (33%)	21 (21.6%)	
＞5	13 (13.4%)	31 (32%)	
TNM stage, n (%)			**0.024**
I-II	25 (25.8%)	16 (16.5%)	
III-IV	20 (20.6%)	36 (37.1%)	
Vascцlar invasion, n (%)			**0.003**
Yes	14 (14.4%)	33 (34%)	
No	31 (32%)	19 (19.6%)	

Supplementary Table 5 Top 20 candidate proteins identified by mass spectrometry

Protein	Unique peptides	Fold change	Annotation
**DYNC1H1**	90	5.64	Cytoplasmic dynein 1 heavy chain 1
**MATR3**	34	5.19	Matrin-3
**SRRM2**	50	5.06	Serine/arginine repetitive matrix protein 2
**CLTC**	50	5.00	Clathrin heavy chain 1
**HLTF**	39	4.87	Helicase-like transcription factor
**SRSF1**	15	4.73	Serine/arginine-rich splicing factor 1
**FASN**	41	4.55	Fatty acid synthase
**PABPC1**	16	4.44	Polyadenylate-binding protein 1
**FUBP3**	25	4.41	Far upstream element-binding protein 3
**HNRNPA3**	22	4.34	Heterogeneous nuclear ribonucleoprotein A3
**PRKDC**	72	4.32	DNA-dependent protein kinase catalytic subunit
**LMNA**	31	4.31	Prelamin-A/C; Lamins are components of the nuclear Lamina
**SYNCRIP**	19	4.24	Heterogeneous nuclear ribonucleoprotein Q
**SKIV2L2**	30	4.17	Superkiller viralicidic activity 2-like 2
**RPL4**	26	4.10	L ribosomal proteins
**SMC1A**	27	4.10	Structural maintenance of chromosomes protein 1A
**ELAVL1**	19	4.10	ELAV-like protein 1
**SMC3**	31	4.06	Structural maintenance of chromosomes protein 3
**RPL6**	18	4.02	60S ribosomal protein L6
**ZCCHC8**	20	4.02	Zinc finger CCHC domain-containing protein 8
